# Development of a fluorescent reporter system for monitoring ER stress in Chinese hamster ovary cells and its application for therapeutic protein production

**DOI:** 10.1371/journal.pone.0183694

**Published:** 2017-08-23

**Authors:** Gargi Roy, Shu Zhang, Lina Li, Eileen Higham, Herren Wu, Marcello Marelli, Michael A. Bowen

**Affiliations:** 1 Antibody Discovery and Protein Engineering, MedImmune LLC, Gaithersburg, Maryland, United States of America; 2 Cell Culture and Fermentation Sciences, MedImmune LLC, Gaithersburg, Maryland, United States of America; Duke University School of Medicine, UNITED STATES

## Abstract

Mammalian cell expression systems have become a workhorse for the production of biotherapeutic proteins. As such, there is an ever increasing demand for higher productivity from these expression platforms to reduce manufacturing costs. While great advances have been made in the optimization of culture conditions and cell line selection to improve productivity, protein mis-folding remains a common limitation to high levels of production of therapeutic proteins. Accumulation of mis- and unfolded protein in the endoplasmic reticulum (ER) causes ER stress and initiates the unfolded protein response (UPR) that results in an activation of protein folding machinery, translation attenuation in an effort to proper folding of the newly synthesized peptides or may even lead to apoptosis if the correct folding is not restored. As a result, UPR associated apoptosis often results in lower protein expression. To better understand the molecular mechanisms in these pathways, we developed a reporter construct that detects Inositol-requiring enzyme 1 (IRE1)-alpha mediated splicing of X-box binding protein 1 (XBP1) to monitor the course of UPR activation in cell lines expressing monoclonal antibodies. Using this reporter we observed a clear activation of UPR in cells treated with known ER stress causing pharmacological agents, such as Tunicamycin (Tm) and Thapsigargin (Tg), as well as in stable IgG expressing cells during fed-batch cultures. Furthermore, we developed a stress metric that we term as ER stress index (ERSI) to gauge basal ER stress in cells which we used as a predictive tool for isolation of high IgG expressing cell lines. This reporter system, with its ability to monitor the stress involved in recombinant protein expression, has utility to assist in devising engineering strategies for improved production of biotherapeutic drugs.

## Introduction

Chinese hamster ovary (CHO) cell lines are the most important industrial mammalian host cell platform for the production of protein biologic drugs [1]. Substantial advancement of bioprocesses in recent years has resulted in highly productive stable cell lines for the manufacture of therapeutic monoclonal antibodies (mAbs). However, the expression of some mAbs and complex multi-specific therapeutic molecules (e.g. bispecific antibodies) remains challenging, despite extensive vector engineering and process improvements. Meeting these expression challenges requires a comprehensive understanding of the various biosynthetic pathways and the burdens imposed by the expression of highly engineered molecules. Folding of nascent polypeptide chains, and the post-translational modifications essential for the maturation of secreted proteins, take place in the ER [2, 3]. Proper function of the ER is perturbed when the influx of nascent polypeptide exceeds the folding capacity [3], which results in the accumulation of misfolded proteins, thereby causing stress and initiation of the unfolded protein response (UPR) [3, 4]. ER stress is an acute condition to protect cells and leads to apoptosis if not properly controlled [5–8]. Common causes for UPR activation during protein production can be due to highly overexpressed target proteins [9], altered metabolic conditions such as glucose deprivation [10], and environmental changes such as hypoxia [4].

UPR consists of three branches of signaling pathways originating from three distinct ER-localized transmembrane signal transducers including activating transcription factor 6 (ATF6), pancreatic endoplasmic reticulum eIF2α kinase (PERK) and inositol requiring endoribonuclease 1 (IRE1) [11]. Accumulation of unfolded proteins triggers the activation of all three pathways. Upon activation, ATF6, a 90 kDa type II transmembrane protein in the ER, is proteolytically cleaved [12], migrates to the nucleus and acts as a transcription activator of ER chaperones such as binding immunoglobulin protein (BiP) and the UPR master regulator X-box binding protein 1 (XBP1) to increase protein folding capacity [13, 14]. PERK, on the other hand, phosphorylates the translation initiation factor eIF2α, causing attenuation of mRNA translation, thus reducing the processing load of nascent polypeptides [15]. Activated IRE1 utilizes its ribonuclease activity and removes a 26 bp intron from XBP1 transcripts, causing a translation frameshift [16, 17], which converts XBP1 into a highly potent transactivator, sXBP1. sXBP1 regulates several UPR target genes including the ER chaperones BiP/GRP78, P58^IPK^ and PDI (protein disulphide isomerase), ER associated degradation components, and various proteins in the secretory pathway [14, 18]. Interestingly, sXBP1 has been shown to play an essential role in terminal differentiation of plasma cells by enhancing the secretory machinery to enable the high productive capacity of these antibody producing cells [16, 19–25].

The central role of UPR components in protein secretion has been studied to characterize cell stress and the effect on protein expression and secretion in CHO cells in manufacturing cell lines. In some approaches, several key components have been overexpressed and shown to impact overall productivity of recombinant protein expression. For example, heterologous expression of the transcription factor XBP1 has been shown to increase stable and transient mAb expression in CHO cells [26–29]. A synthetic monitoring system containing major promoter *cis*-elements responding to ATF6 and XBP1 has been designed to detect activation of UPR in large scale protein production [30]. Another study described a GFP reporter system using the GRP78 promoter and showed its usefulness over other ER stress element promoters such as CALR, GRP94, and XBP1 in the identification of high expresser cells [31]. However, these reports show minimal data-driven insight into how the activation of UPR directly impacts recombinant IgG expression. In order to bridge the knowledge gap and gain better understanding into the effect of ER stress in cells capable of high level protein expression, we designed a novel dual fluorescent protein reporter system utilizing the splicing of XBP1 as a marker for UPR activation. In designing the reporter, we focused on the transactivating role of sXBP1 for a comprehensive understanding of UPR activation that influences the secretory pathway machinery for efficient IgG secretion. This reporter allowed us to develop an ER stress index (ERSI) measurement to gauge stress levels and compare different mAb expressing cell lines. The ERSI is shown to be a good predictor of productivity and was further validated with a detailed pathway analysis of UPR activated genes and proteins to better understand the signaling cascades that are activated during mAb expression. Furthermore, we applied the dual fluorescent protein reporter system to cell line development to improve the identification of cell lines with higher mAb productivities.

## Materials and methods

### Construction and expression of a UPR dual fluorescent reporter system

A dual fluorescent reporter gene encoding a RFP and GFP fusion separated by an XBP1 splice sequence was chemically synthesized (GeneArt, Thermo Fisher Scientific, Carlsbad, CA) and cloned into mammalian expression vector pcDNA3.1 Hygro (Thermo Fisher Scientific, Carlsbad, CA) at HindIII/BamHI sites downstream of the CMV promoter. Stable CHO cell lines containing the dual fluorescent reporter were selected in CD-CHO medium (Thermo Fisher Scientific, Carlsbad, CA) supplemented with 500μg/ml Hygromycin B (Thermo Fisher, Carlsbad, CA). Cell lines expressing the dual fluorescent reporter were screened by flow cytometry (BD LSRII, BD Biosciences, San Jose, CA); data analysis was performed using FlowJo software (Tree Star Inc, Ashland, OR).

### Cell culture and monitoring of ER stress

Experiments were performed using suspension CHO cells derived from CHOK1 adapted to grow in serum-free medium. mAb-expressing clonal CHO cell lines containing the dual fluorescent reporter were routinely sub-cultured as suspension cells in proprietary in-house production medium under the appropriate selection pressure. Cells were grown in E125 flasks and sub-cultured every 3 or 4 days to a viable cell seeding density of 0.5 × 10^6^ cells/mL in 30 mL in a Multitron incubator (ATR Biotech, Laurel, MD) at 37°C in presence of 6% CO_2_, with 140 RPM and 80% relative humidity. For activation of ER stress using Tunicamycin, 10μM (Sigma, St. Louis, MO) and Thapsigargin, 300nM (Sigma, St. Louis, MO) cells were seeded in 6 well plates at a density of 0.5E6/ml 24 hours prior to the cell treatment. Tunicamycin (Tm) and Thapsigargin (Tg) were dissolved in DMSO while DMSO treated cells were used as vehicle control. Cells were harvested at different time points such as 3h, 6h, 9h, 18h and 24h post treatment and were analyzed by flow cytometry to monitor the expression of dual fluorescent protein (RFP-GFP) as an indicator for induction of UPR. Harvested cell pellets at different time points were used for RT PCR and western blot analyses to monitor the status of key UPR pathway proteins. Activation of UPR was also monitored in stable mAb expressing cell lines during a14-day fed-batch assay carried out in duplicate by using MedImmune proprietary medium and feed regimens. Cell sampling for cell performance monitoring, IgG quantitation, ERSI determination by flow cytometry, and expression analysis by Western blotting and qRTPCR were performed on days prior to addition of nutrient feed.

### Western blot analysis

Cells were lysed in M-PER mammalian protein extraction reagent containing Halt Protease (Pierce Biotechnology, Rockford, lL) following the manufacturer’s recommendations. Twenty micrograms of proteins from these cell free extracts were analyzed on SDS-PAGE (Bio-Rad, Hercules, CA) and transferred to nitrocellulose membrane using Trans-Blot Turbo transfer system (Bio-Rad, Hercules, CA). Membranes were probed with rabbit anti-BiP, anti-PDI, anti-p-PERK, anti-eIF2α, anti-P-eIF2α, anti-P58^IPK^ (Cell Signaling, Boston, MA, USA), mouse anti-ATF-6 (Pierce Biotechnology; Rockford, lL, USA), and rabbit anti-XBP1 (Abcam, Cambridge, MA, USA). Antigen specific bands were detected by incubation of membranes using HRP-conjugated anti-rabbit or anti-mouse antibodies followed by incubation with SuperSignal West Pico Chemiluminescent Substrate (Pierce Biotechnology, Rockford, lL, USA) and image analysis by ImageQuant LAS4000 (GE Heathcare Bio-Sciences, Pittsburgh, PA, USA).

### RTPCR analysis and DNA sequencing

Total RNA was isolated using an RNeasy plus mini kit (Qiagen), and reverse transcription (RT) was performed using TaqMan Reverse Transcription Reagents (Thermo Fisher Scientific, Grand Island, NY) with 1.0 μg RNA according to the manufacturer’s protocol. Splicing of XBP1 was detected by PCR amplification by denaturing at 95°C for 30 seconds, followed by annealing at 55°C for 1 minute and extension at 72°C for 30 seconds, for 40 cycles using target specific primers XBP1-F1 and XBP1-R1. The amplified cDNA products were analyzed on Agilent DNA 7500 chips using a Bioanalyzer (Agilent Technologies, Santa Clara, CA). The spliced variant of XBP-1 (sXBP-1) was distinguished by a 26nt size difference. Primers for the qPCR assays are listed in Table 1. Light chain (LC), heavy chain (HC) and glyceraldehyde-3-phosphate dehydrogenase (GAPDH) mRNA expression levels were measured using TaqMan technology on the 7900HT Fast Real-Time PCR System (Thermo Fisher Scientific, Grand Island, NY). The probes and primers were generated by assays-by-design (Applied Biosystems, Foster City, CA). The probes contained a 6-carboxy-fluoresceinphosphoramidite (FAM dye) label at the 5' end of the oligo and a nonfluorescent quencher at the 3' end. QRTPCR was performed by denaturing at 95°C for 20 seconds and then cycling at 95°C for 1 second and 60°C for 20 seconds, for 40 cycles. CHO-specific glyceraldehyde-3-phosphate dehydrogenase (CHO-GAPDH) was used as the endogenous RNA control. Expression levels of LC and HC were normalized to endogenous CHO-GAPDH. EDEM1, HERPUD1, Derlin1, CHOP, ATF4 and GAPDH mRNA expression levels were measured using CHO specific primers listed in Table 1 using SYBR Green Real-Time PCR Master Mix utilizing the 7900HT Fast Real-Time PCR System (Life Technologies, Grand Island, NY, USA). qPCR was carried out by denaturing at 95°C for 20 seconds and then cycling at 95°C for 1 second and 60°C for 20 seconds, for 40 cycles. CHO specific glyceraldehyde-3-phosphate dehydrogenase (GAPDH) was used as the endogenous RNA control. Expression levels were normalized to endogenous GAPDH.

**Table 1:** Primers used for real-time RTPCR for mRNA expression analysis of UPR pathway genes.

Primer Name	Target Gene	Primer Type	Primer Sequence
XBP1 F1	XBP-1	Forward	5'- cacctgagcccggaggag-3'
XBP1 R1	XBP-1	Reverse	5'- gagttcattaatggcttccagc-3'
RFP F2	RFP	Forward	5′-ccctcgtcagggaatcttgaagggcg-3′
GFP R2	GFP	Reverse	5′-ggcgcctttggtgctcttcatcttgttgg-3′
ATF4 F1	ATF4	Forward	5′-attctccgggacagactgga-3′
ATF4 R1	ATF4	Reverse	5′-tggccaattgggttcactgt-3′
EDEM1 F1	EDEM1	Forward	5′-acgtcttcggaattgcctt-3′
EDEM1 R1	EDEM1	Reverse	5′-gtcgatctggcgcatgtaga-3′
HERPUD1 F1	HERPUD1	Forward	5'-ccacttgagccgagtctacc-3'
HERPUD1 R1	HERPUD1	Reverse	5'-cagcaccctttgttttggct-3'
CHOP F1	CHOP	Forward	5'-cttcgttatccccggctctc-3′
CHOP R1	CHOP	Reverse	5′-gactcagctgccatgtctgt-3′
DERLIN1 F1	DERLIN 1	Forward	5′-catcggggactggttcagga-3′
DERLIN1 R1	DERLIN 1	Reverse	5′-tggaagcgatagaggaaggc-3′
CHO-GAPDH F1	GAPDH	Forward	5′-tgcccagaacatcatccctg-3′
CHO GAPDH R1	GAPDH	Reverse	5′-caggcgacatgtcagatcca-3′

### Confocal imaging

Confirmation of activation of UPR using the dual fluorescent reporter by expression of GFP-RFP fusion protein was performed by visualization and imaging by confocal microscopy using a Leica SP5 confocal microscope (Leica Microsystems Inc. Buffalo Grove, IL).

### Transient expression of RXG (RFP-XBP-1-GFP) reporter in stable IgG expressing cell lines

Basal ERSI in stable IgG expressing cell lines was determined by transient expression of the RXG reporter plasmid using 96 Well Shuttle System (Lonza Biologics, Walkersville, MD) followed by flow cytometry in high throughput format using an LSRII instrument (BD Biosciences, San Jose, CA). Basal ERSI is the measure of ER stress while the cells are in maintenance culture, and not in fed-batch culture. ERSI was calculated from the statistics generated by FlowJo software (TreeStar Corporation, Ashland, OR) using the formula described in the Results.

## Results and discussions

### Validation of a dual fluorescent reporter construct as an indicator of cell stress

**Fig 1:**
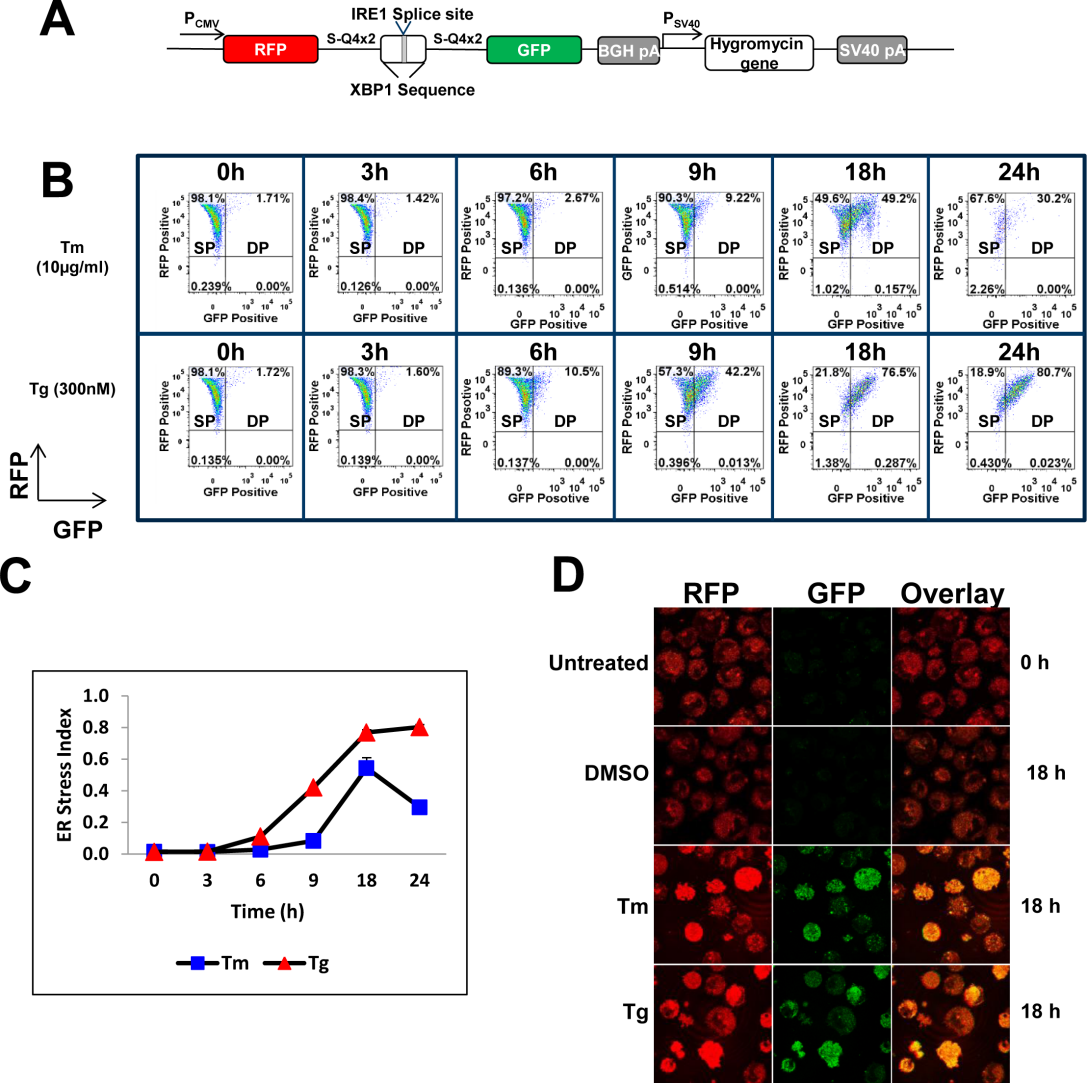
Design of the RXG reporter system for the detection of cell stress by treatment with Tm and Tg. (A) Dual fluorescent reporter construct encoding RFP and GFP separated by a XBP-1 splice site. Efficient splicing by p-IRE1 causes a frameshift that places GFP in frame with RFP. (B) Cell line 10-1RXG20 stably expressing the reporter and IgG was treated with 10 μg/mL Tunicamycin (Tm) or 300 nM Thapsigargin (Tg). Cells were analyzed by flow cytometry at the indicated times after treatment. SP and DP refer to single positive (RFP) and double positive (RFP-GFP) cell populations respectively. At 24h post treatment with Tm, cell morphology changed due to the trigger of apoptosis and much of the cell population were outside of the live gate population showing the lack of viable cells at 24h time point. (C) The ER stress index (ERSI) was calculated for each time-point following treatment with Tm and Tg. Both treatments elicit ER stress, but with different kinetics. (D) Confirmation of dual fluorescent protein expression due to cell treatments with Tm and Tg by confocal microscopy. No GFP expression was observed in untreated cells or the vehicle treated cells.

ER stress has been shown to result in splicing of XBP1 by phosphorylated IRE1 (p-IRE1) to generate sXBP1 [14]. In an effort to monitor ER stress, we utilized a reporter construct encoding a RFP-GFP fusion, where the coding sequences for red (RFP) and green fluorescence protein (GFP) are separated by a stretch of XBP1 sequence containing a 26nt IRE-1 responsive splice site (**R**FP-**X**BP-1-**G**FP; RXG, see Fig 1A). Thus, only RFP is expressed in the absence of UPR, but with UPR activation the reporter mRNA is spliced, bringing the RFP and GFP into the same translational reading frame, resulting in the expression of a dual fluorescent protein that can be monitored by flow cytometry.

Cell treatment with pharmacological agents such as Thapsigargin (Tg), a non-competitive inhibitor of sarco endoplasmic reticulum Ca2^+^ ATPase [32], and Tunicamycin (Tm), an inhibitor of N-linked glycoproteins [33], has been widely used to elicit ER stress and activation of the UPR pathway proteins. To test whether the RXG reporter functioned as an indicator of cell stress, the dual fluorescent reporter plasmid was first stably expressed into an IgG expressing cell line, 10–1, to generate cell line 10-1RXG20. Actively growing 10-1RxG20 cells were treated with Tm (10μg/ml) or Tg (300nM). Flow cytometry was used to quantify the expression of RFP and GFP in cells to assess the level of splicing as a functional readout of cellular stress (Fig 1B). For both treatments, we observed a gradual increase in the RFP-GFP double positive (DP) cell population over the time course of treatment starting from the untreated 0hr population indicating a successful proof of concept (Fig 1B–1D). The onset of ER stress detected by the dual FL reporter was specific to Tm and Tg treatments since the vehicle (DMSO) treated controls showed no GFP expression as determined by flow cytometry (S1A Fig). In an effort to quantify the level of ER stress we examined the ratio between double positive and single positive (SP; expressing only RFP) cells. The metric described by the equation ERSI = DP/(SP+DP) was termed ER stress index (ERSI) and reflects a measurement of the level of stress within a cell population. The onset of ER stress was evidenced by plotting the ERSI kinetics (Fig 1C). In both cases an elevated ERSI was observed, albeit Tm treatment showed a delay in the onset of stress relative to Tg. The onset of ER stress due to cell treatments by Tm and Tg, indicated by the flow cytometric detection of dual fluorescent reporter, was further confirmed by confocal microscopy where treated cells showed both RFP and GFP expression whereas GFP expression was not detected in untreated and vehicle (DMSO) treated cells (Fig 1D).

**Fig 2:**
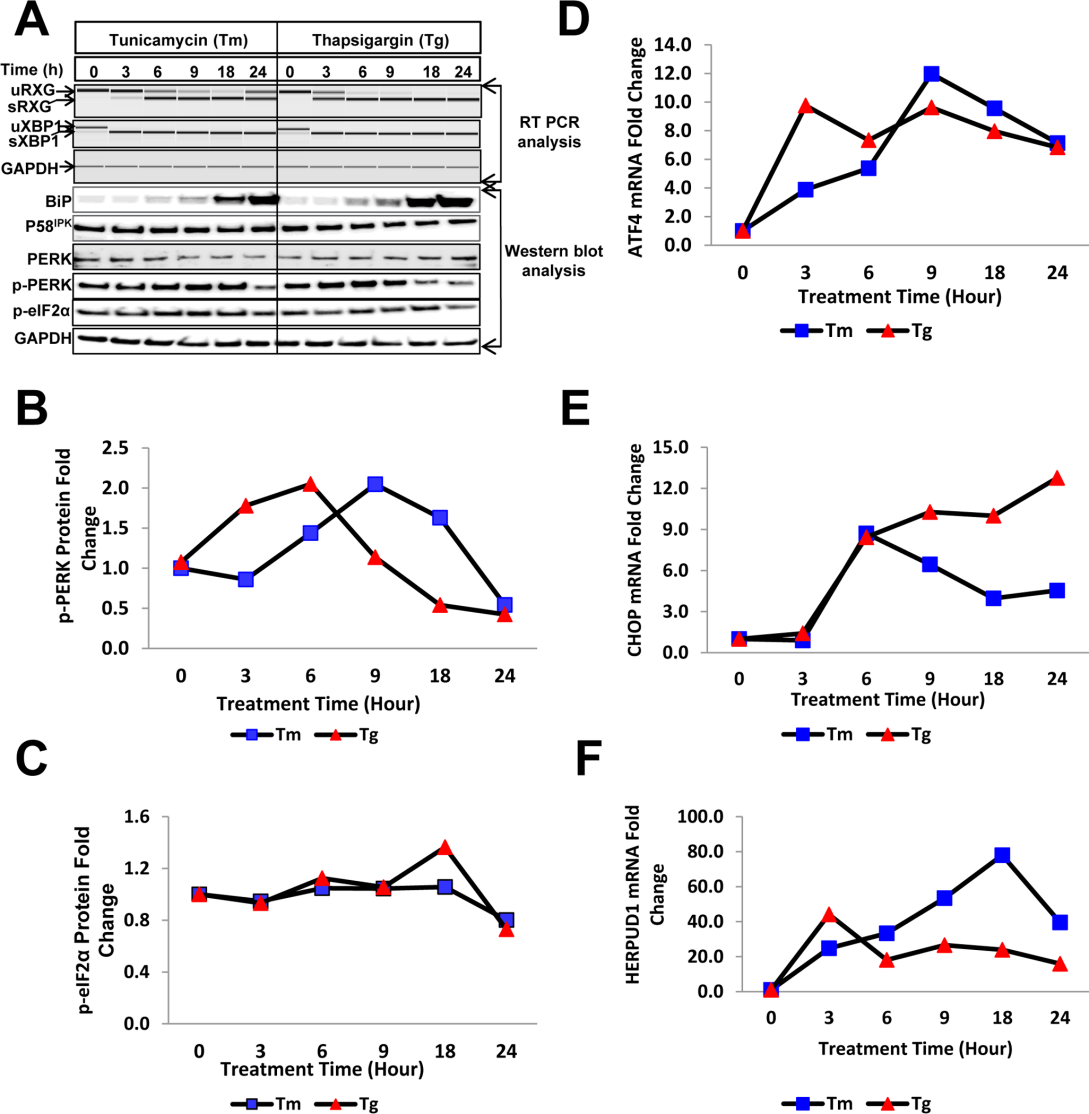
Validation of the RXG reporter system for the detection of cell stress by treatment with Tm and Tg by expression analysis of ER stress proteins. (A) The kinetics of IRE1-mediated splicing of the synthetic reporter (sRXG) and endogenous XBP1 (sXBP1) were determined using RTPCR, followed by electrophoretic analysis (top panels) with GAPDH used as a loading control. Lysates from cells treated with Tm (10 μg/mL) and Tg (300 nM) were used for immunoblotting using antibodies directed against BiP, P58^IPK^, PERK, phosphorylated PERK, phosphorylated eIF2α and GAPDH. (B) Quantitation of p-PERK and (C) p-eIF2α proteins determined by Western blot analysis at 0–24 hours post cell treatments with Tm (10 μg/mL) and Tg (300 nM). (D) Kinetics of ATF4 and CHOP (E) mRNA activation measured by real-time qPCR following cell treatments with Tm and Tg. (F) Quantitation of HERPUD1 mRNA upon cell treatments with Tm and Tg.

The splicing of XBP-1 and the RXG reporter was confirmed by RTPCR followed by electrophoretic analysis using an Agilent Bioanalyzer (Fig 2A). The PCR products for both endogenous XBP1 and RXG reporter represent unspliced (uXBP1 and uRXG) and spliced (sXBP1 and sRXG) variants (Fig 2A, top panel). The kinetics of the splicing of the reporter mRNA (Fig 2A, top panel) corresponded well with the shift in the cell population determined by flow cytometry in Fig 1B between 0 to 24 hours post drug treatment and the ERSI analysis (Fig 1C) for the same cultures. Interestingly, we observed that endogenous XBP1 was spliced within 3 hours post drug treatment whereas the RXG splicing showed slower kinetics. This might be due to the low abundance of endogenous XBP1 mRNA readily spliced by p-IRE1 compared to the highly abundant synthetic reporter driven by a CMV promoter. A complete conversion of uRXG or uXBP1 to respective spliced variants was specific to Tm and Tg treated cells in contrast to the untreated and DMSO treated cells where splicing can be found at a much lesser extent (S1B Fig, top panel).

Expression analysis of key ER stress proteins revealed significant accumulation of BiP starting from 6 hours post cell treatment with both Tm and Tg, while the P58^IPK^ expression remained unchanged (Fig 2A). The induction of BiP was specific to Tm and Tg treatments as its expression levels remained unchanged in untreated and vehicle (DMSO) treated controls (S1B Fig, bottom panel). The PERK pathway was activated as evidenced by decreased expression of native PERK and a 2-fold induction of p-PERK was observed between 6–18h post treatment with Tm. A similar 2-fold induction of p-PERK in Tg treated cells was observed between 3–9 h post treatment while native PERK remained more or less unchanged (Fig 2A and 2B). However, eIF2α, a component downstream of p-PERK, was only minimally phosphorylated between 6 and 18h after treatment (Fig 2A and 2C) in these cells, and not within 30 min of treatment as previously reported [33]. Nevertheless, the modest increase of p-eIF2α was sufficient to trigger the activation of ATF4 and CHOP mRNA (Fig 2D and 2E) associated with cell death via BCl2 activation [8]. A significant activation of HERPUD1, a protein linked with the ER associated degradation (ERAD) pathway responsible for maintaining the ERQC (Endoplasmic Reticulum quality control) was also observed (Fig 2F). In contrast, the low basal expression levels for CHOP, ATF4 (S1C Fig) and HERPUD1 (S1D Fig) were maintained in the untreated and the vehicle (DMSO) treated cells. Interestingly, the early increase in the ERSI of Tg- relative to Tm-treated cells was also observed in the analysis of stress response proteins (P-PERK and ATF4) and suggest that the dual fluorescent reporter construct can be used as an indicator of ER stress in cells when exposed to known pharmacological agents like Tm and Tg.

### Detection of UPR activation in recombinant IgG expressing cells using the dual fluorescent reporter

**Fig 3:**
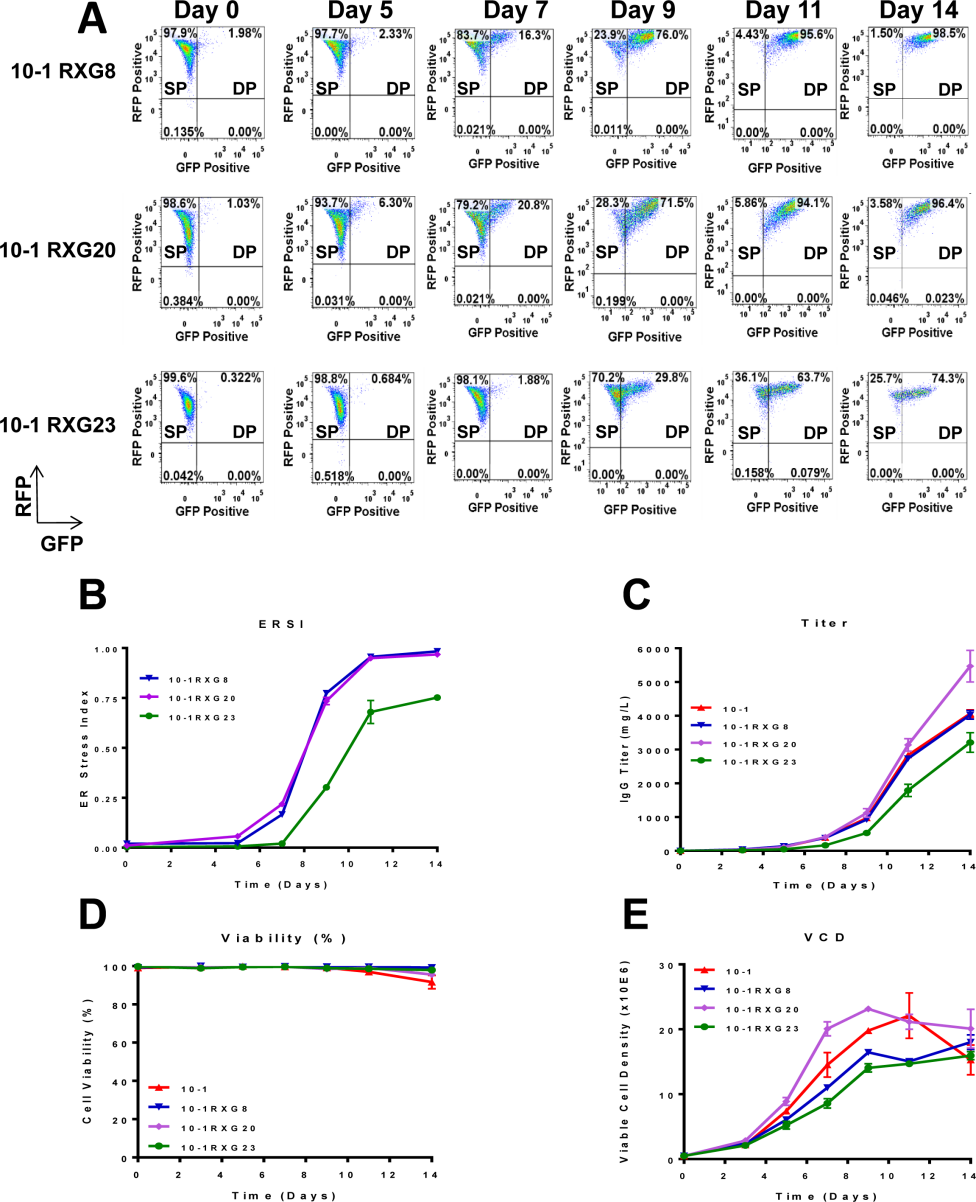
Determination of ER stress using dual FL reporter in IgG expressing cells in fed-batch cultures. (A) The RXG reporter was utilized to assess cell stress in IgG-expressing cells (10-1RXG8, 10-1RXG20 and 10-1RXG23) during a 14-day fed-batch culture and cells were monitored for RFP and GFP expression by flow cytometry. Accumulation of double positive (DP) cells increased over time during the production phase in the 14-day fed batch process. (B) Graphical representation of ERSI, IgG titer (C), cell viability (D) and viable cell density (E) during the 14 day fed-batch run. The ERSI more or less paralleled the productivity of the cultures.

Having established the RXG reporter as an indicator of cell stress, we tested whether stable cell lines producing different levels of IgG exhibited distinguishable signatures of stress over the course of a fed-batch culture. Three independent cell lines derived from 10–1 by transfection with the RXG reporter and with different productivity levels (10-1RXG8, 10-1RXG20, and 10-1RXG23) were subjected to a 14-day fed-batch culture. RFP and GFP expression was monitored by flow cytometry in each culture together with the parental line 10–1 lacking the RXG reporter. In all RXG reporter cell lines, the population shifted from SP to DP starting on day seven and continuing over the course of the culture indicating increasing XBP1 splicing activity. Representative scatter plots for 10-1RXG8, RXG20 and RXG23 are shown in Fig 3A. Using these data, the ERSI values were calculated for the 10-1RXG8, 10-1RXG20, and 10-1RXG23 cultures and plotted (Fig 3B). The basal ERSI values for 10-1RXG8, 10-1RXG20, and 10-1RXG23 were 0.020, 0.010 and 0.003, respectively, but rose sharply starting on day seven and reached its peak by day 11 in all cases (Fig 3B). Interestingly, the rise in the ERSI paralleled the production kinetics of IgG titer (Fig 3C), so that the most productive cell lines also have higher ERSI scores. Notably, the lowest producing cell line RXG23 showed the lowest basal ERSI, a delayed rise in ERSI and reached a lower maximal ERSI value compared to the other cell lines. The difference in productivity of the cells was not due to loss of cell viability (Fig 3D), but differences in viable cell density (VCD) were observed (Fig 3E) with the lowest expresser 10-1RXG23 reaching the lowest VCD in contrast to the top expresser 10-1RXG20.

**Fig 4:**
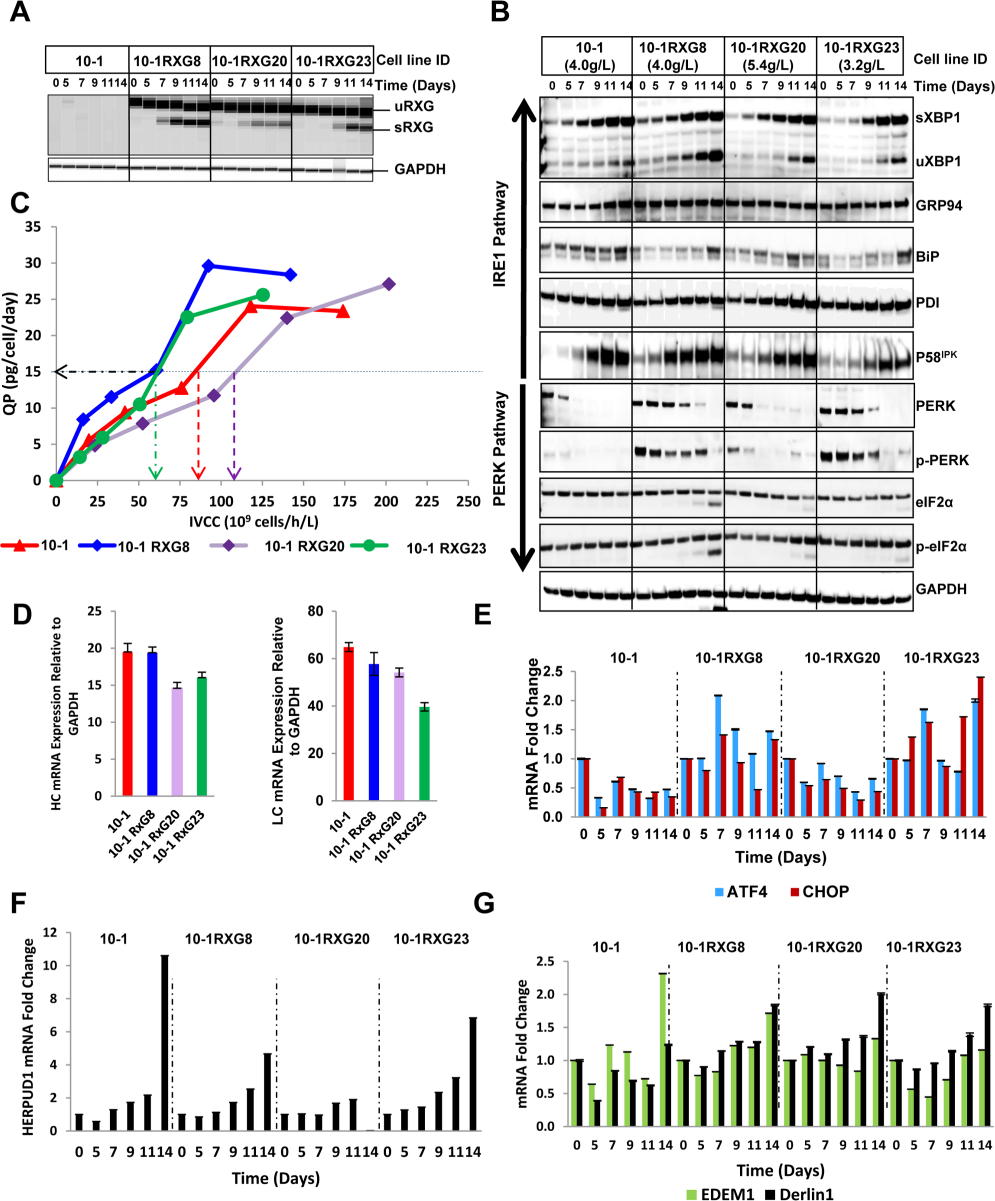
Expression analysis of UPR pathway proteins and monitoring with the RXG reporter in IgG-producing cell lines. (A) Splicing of the RXG reporter (sRXG) was determined by RTPCR followed by analysis using DNA 7500 chips with an Agilent Bioanalyzer. (B) Detection of key proteins involved in UPR by Western blot analysis. IRE1 pathway proteins XBP1 (uXBP1) and its spliced form (sXBP1), chaperones GRP94, BiP, PDI, P58^IPK^, and PERK pathway proteins native PERK and eIF2α and their phosphorylated forms (p-PERK and p-eIF2α) were detected by Western blot analysis. GAPDH was used as a loading control. (C) Kinetics of QP and IVCC in IgG expressing cells during fed-batch culture. Threshold metrics for QP and IVCC critical for sustained inhibition of p-PERK are marked by dashed arrowed lines. (D) HC and LC mRNA expression of multiple RXG expressing stable IgG cell lines relative to GAPDH determined by qRTPCR. (E) Kinetics of ATF4 and CHOP mRNA expression during fed-batch culture of IgG expressing cells normalized on day 0 and expressed relative to endogenous GAPDH determined by real-time Q RTPCR. (F) Kinetics of ERAD and ERQC pathway proteins such as HERPUD1, (G) EDEM1 and Derlin1determined by real-time QRTPCR during fed-batch culture of IgG expressing cells.

The differences in ERSI, titer and VCD led us to further investigate the state of ER stress in these cells. First, we examined the kinetics of UPR activation by quantifying the splicing of the XBP1 sequence present within the RXG reporter by RTPCR analysis (Fig 4A). Increasing amounts of sRXG starting from day seven were evident in the more productive cell lines, 10-1RXG8 and 10-1RXG20 (Fig 4A). In contrast, RXG mRNA splicing was not observed until day 9 in 10-1RXG23 (Fig 4A). This delay corresponded well with both the slower ERSI kinetics (Fig 3B) and IgG production kinetics of this cell line (Fig 3C) and may be due to a delay in the UPR activation in the lower producing cells. In an effort to characterize the components of the pathway regulating the UPR we monitored the levels of expression for a panel of key proteins associated with UPR by Western blotting over the course of a fed-batch culture (Fig 4B). As expected, we observed increasing XBP1 expression and splicing (sXBP1) that closely matches the RXG reporter kinetics (Fig 4A). Increasing expression of p58^IPK^, and PDI was observed in all cell lines while GRP94 maintained uniform expression throughout the time course with an exception in parental cell line 10–1 where an induction was seen on days 12 and 14. BiP expression was generally slightly induced across the time points in all the cell lines. In each case, 10-1RXG23 showed a delayed response relative to the more productive cell lines. Interestingly, sustained expression of p-PERK, activated form of PERK, was observed in 10-1RXG8 and 10-1RXG23 and is consistent with the reduced VCDs observed in these cultures. The more productive cells, 10-1RXG20 and 10–1, showed an inhibition of PERK and p-PERK.

As the activation of PERK pathway can lead to a reduction in expression by translation attenuation regulated by phosphorylated eIF2α, we examined specific expression of antibody in each of these cell lines. For each cell line the specific productivity (QP) was calculated and plotted against the integrated viable cell concentration (IVCC) of the culture (Fig 4C). From these data, we observed that both 10-1RXG8 and 10-1RXG23 reached a QP of 15 much earlier than the other cell lines. These cells also showed increased levels of PERK and p-PERK suggesting that a threshold QP of < 15 PCD (pg/cell/day) and IVCC of >60x10^9^ cells/h/L by day 9 might be a metric in these suspension CHO cell lines for sustained activation of p-PERK and the downstream proteins leading to apoptosis. However, the simultaneous induction of the chaperone P58^IPK^ that may function to increase folding capacity and help cope with the increased influx of nascent polypeptides contributing to a relatively higher final IgG titer (4.0g/L) for 10–1 RXG8 compared to 10-1RXG23 (3.2g/L). It is noteworthy that p-eIF2α remained elevated in 10-1RXG23 throughout the production time course which might have maintained some degree of translation halt causing an overall lower productivity than10-1RXG8. However, sustained p-PERK expression in 10-1RXG8 did not have an impact on p-eIF2α or its downstream signaling cascade. In summary, in 10-1RXG23 a combination of events such as a delayed UPR activation, a delayed P58^IPK^ induction, an early rise in QP with low IVCC, sustained p-PERK expression, and increased p-eIF2α, might have caused an intermittent halt in the protein translation, resulting in overall low productivity.

Interestingly, the low productivity of 10-1RXG23 was not due to low expression of the HC. RT-PCR analyses of HC mRNA showed similar expression levels in 10-1RXG20 and 10-1RXG23 (Fig 4D). However, a 1.4-fold higher expression of LC was seen in 10-1RXG20 and about 1.6-fold higher final productivity was achieved in this cell line (5.2g/L), possibly due to better assembly of HC and LC compared to 10-1RXG23. The lower productivity of 10-1RXG23 may also be due to contrasting combination of ER stress signaling events as discussed above, potentially caused by the excess HC. In such a scenario, P58^IPK^ may be performing a dual function as a chaperone as well as an inhibitor of the PERK/eIF2α/ATF4 pathway [34]. Indeed, 10-1RXG23 and 10-1RXG8 showed elevated levels of p-eIF2α, ATF4 and CHOP relative to 10–1 and 10-1RXG20 cell lines (Fig 4E). However, the level of induction was significantly lower than the Tm- and Tg-treated cells (Fig 2D and 2E) and was not sufficient to trigger apoptosis in these cell lines as cell viability remained high in all cultures (Fig 3D). Additionally, the ERAD pathway proteins such as HERPUD1 (Fig 4F), EDEM1 and Derlin1 (Fig 4G) remained minimally induced during the expression kinetics (Fig 3C). Overall, these data suggest that a perfect balance was maintained between the different arms of ER stress pathways resulting in induction of chaperones aiding the proper folding and efficient secretion of IgG. In summary, the correlation between the endogenous XBP1 splicing or the mRNA splicing of the reporter and the IgG production kinetics indicate that UPR plays an important role in IgG expression and secretion. The data also indicate a connection between the timing of the XBP1-mediated UPR activation, induction of chaperones and the final IgG titer.

### Application of the dual fluorescent reporter system to a stable cell line development campaign

**Fig 5:**
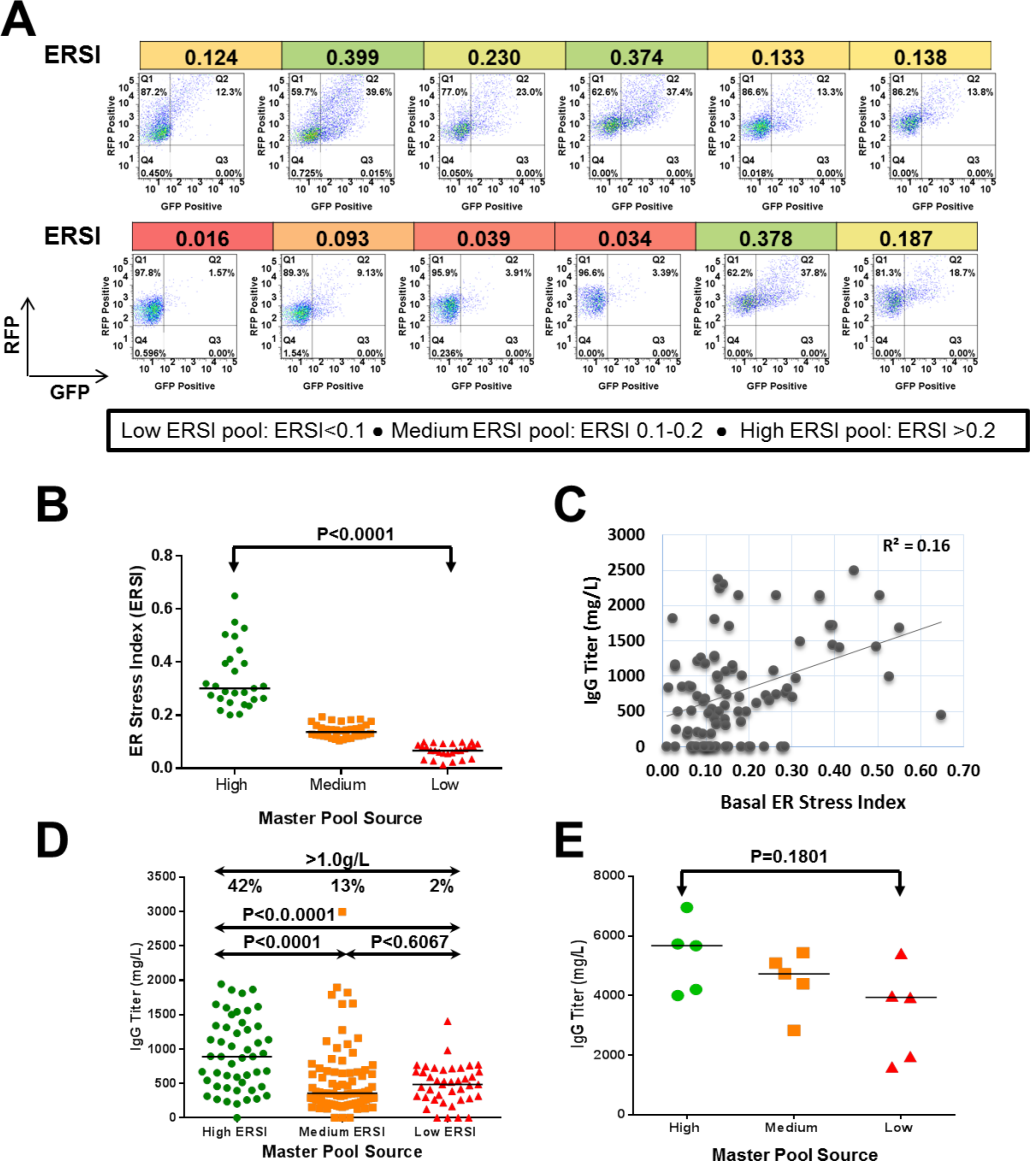
The dual fluorescence reporter and ERSI can be used to enrich for high producers in a stable cell line development campaign. (A) The dual fluorescent RXG reporter was transiently transfected into mAb-expressing primary isolates, and then RFP and GFP levels quantified by flow cytometry 48 h. post-nucleofection. Scatter plots for a representative set of isolates are shown. (B) ERSI distribution for 90 mAb-expressing primary isolates grouped into high, medium and low basal ERSI master pools. The differences in median ERSI between high and medium and high and low ERSI master pools were statistically significant (P<0.0001) as determined by Unpaired t test. (C) Correlation plot between the basal ERSI and the IgG titers of the primary isolates. (D). Titer distribution of mAb clonal cell lines determined by fed-batch in multi-well plates. The median titers of the clones derived from high and medium or high and low ERSI master pools are statistically significant as determined by Unpaired t test (P<0.0001), however, the median titer in clones derived from medium and low ERSI master pools were not statistically different (P = 0.6067). 42% clones derived from high ERSI master pools produced >1.0g/L, whereas only 13% and 2% clones derived from the middle and the low ERSI master pools respectively, expressed higher than 1.0g/L IgG. (E). Titer distribution of top clones from panel C during fed-batch in shake flasks. Isolates with higher initial ERSI scores also generated higher titers. The highest producer clone was derived from with top clone high ERSI master pool reaching up to 6.9g/L. Statistical parameter such as Unpaired t test was used to determine the P value (P = 0.1801) to compare the titer range of the top clones derived from the high and low ERSI master pool sources.

The observation that XBP-1 splicing correlated with improved titer has been previously described [27, 31]. In order to determine whether there was a correlation between basal ER stress and IgG expression levels, we introduced the dual fluorescent reporter transiently into 90 stable cell lines derived during a typical mAb CLD campaign. The relative transfection efficiency across the cell lines was >99% based on the RFP expression of the reporter. The basal ERSI value for each cell line was determined by high throughput flow cytometry and representative cell line scatter plots are shown in Fig 5A. Based on the calculated ERSI value, cell lines could be placed into three distinct groups. Primary isolates with ERSI values lower than 0.1 were categorized under the low ERSI group, ERSI values of 0.1–0.2 were in the medium ERSI group and the isolates containing ERSI values of >0.2 were grouped as high ERSI (Fig 5B). In a conventional CLD campaign, the expressing colonies are combined in a single pool prior to single cell sorting by using a flow activated cell sorter (FACS). [35]. However, in this case, the colonies within each ERSI category were combined into independent master pools and then cloned by FACS to generate single cell clones in microtiter wells. A total of 171 clones derived from high, medium and low ERSI master pools were screened for IgG expression in multi-well fed-batch culture. Although there was a weak correlation (R square of 0.16) found between the basal ERSI and the IgG titer of the of the parent primary isolates (Fig 5C), a higher frequency (42%) of high titer clones (>1.0g/L) were derived from the high ERSI master pools compared to medium (13%) and low ERSI (2%) master pools (Fig 5D). This trend was also maintained when the top clones from each ERSI master pool were subsequently analyzed in a 14-day fed-batch assay in shake flasks (Fig 5E).

**Fig 6:**
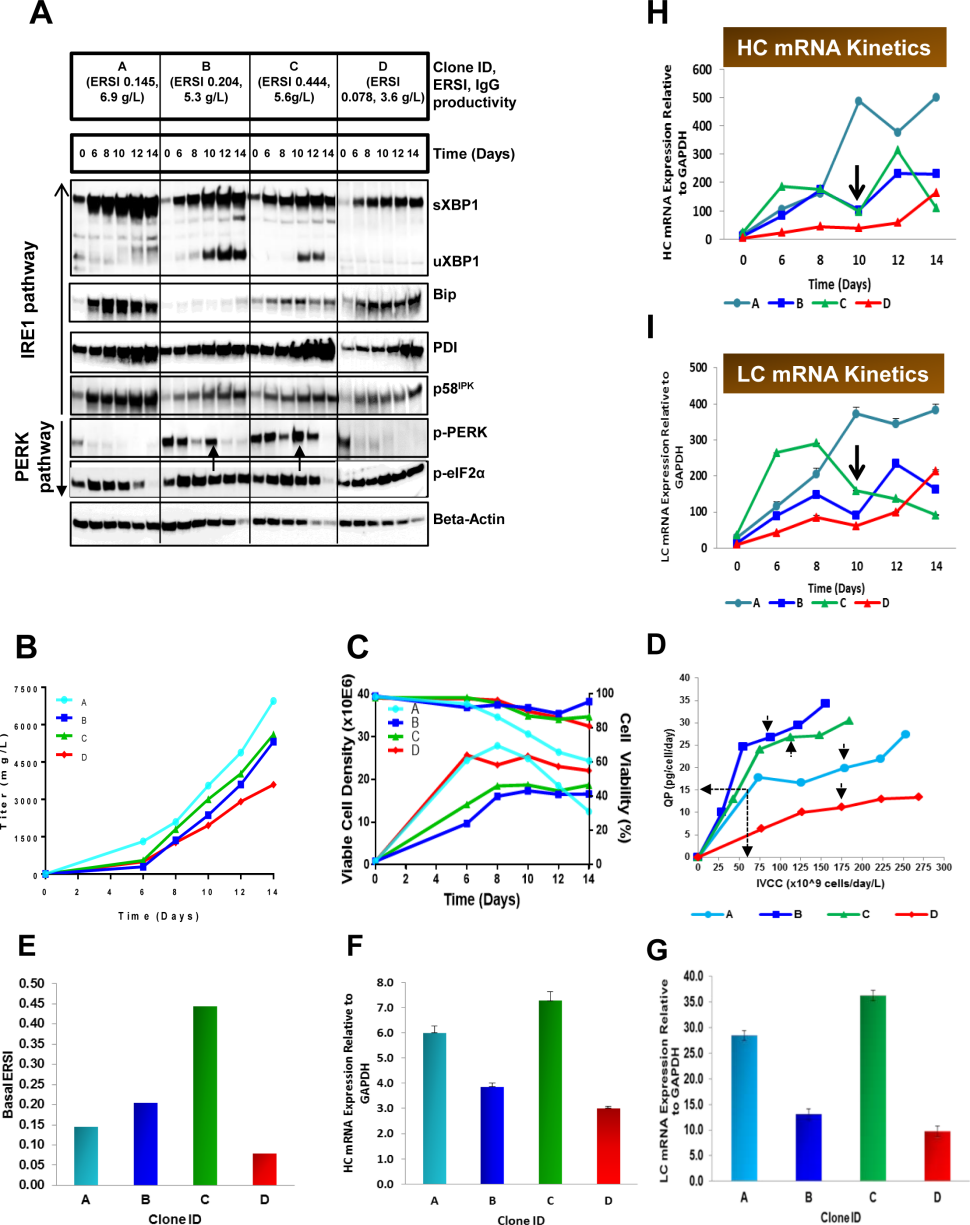
Tracking the UPR activation in IgG expressing cells by biochemical analyses. **(A)** Stable clones with different demonstrated titers of a target IgG were examined for differential expression of components of the IRE-1 and PERK pathway by Western blotting. The high producing clone A showed elevated BiP, PDI, p58^IPK^ and reduced p-PERK levels relative to other clones with lower productivity (clones B, C and D). An arrow indicates up regulation of activated PERK on day 10 caused by the early rise in QP in clones B and C. (B) IgG expression titers during 14-day fed-batch culture. **(C)** Cell performance kinetics determined by cell viability and VCD during 14-day fed-batch culture. **(D)** Kinetics of QP and IVCC of IgG expressing clones A, B, C and D during 14-day fed batch culture. Threshold metrics for QP and IVCC critical for sustained inhibition of p-PERK are marked by dashed arrow lines. **(E).** Determination of basal ERSI in clones A, B, C and D. **(F).** Basal HC expression by real-time PCR in different IgG expressing clones. **(G)** Determination of basal LC expression by real-time PCR in different IgG expressing clones. **(H).** HC and **(I)** LC mRNA kinetics over the course of a 14 day fed-batch culture assay. Down arrow indicates drop in HC and LC mRNA levels in clones B and C between days 8 and 10.

To gain some insight into this effect, four different clones with different final productivities ranging from 6.9g/L to 3.6g/L, derived from the high (clones A, B and C) and low (clone D) ERSI master pools were examined for the expression levels of relevant UPR associated proteins over the course of a 14 day fed-batch culture. Increased expression and splicing of XBP1 and elevated expression of chaperones, such as BiP, PDI and P58^IPK^, were observed in the top expresser (clone A; 6.9g/L), whereas the lower producer (clone D; 3.6g/L) showed substantially lower expression of these same proteins (Fig 6A). The other high producing clones (B and C) showed sustained activation of p-PERK as well as elevated PDI. These data suggest that different strategies may be adopted by cells to cope with the demands of high titer IgG expression. The titer plots for these four clones are shown in Fig 6B. Sustained activation of p-PERK in clones B and C could very well be correlated with the previously mentioned threshold of QP and IVCC. It is evident that the peak VCD (Fig 6C) is much lower in clones B and C (<20x10^6^/ml) compared to clones A and D (>25 x10^6^/ml) and indeed, in both of these clones the QP reached almost 25 pcd (pg/cell/day) with an IVCC of about 100x10^9^cells/h/L by day 10. In contrast clones A and D showed a slower rise in per cell productivity; reaching a QP of only19 and 11 respectively at IVCC reaching 150x10^9^cells/h/L (Fig 6D). Interestingly, clone C exhibited the highest basal ERSI score (0.444) (Fig 6E), the highest HC (Fig 6F) and LC basal mRNA levels (Fig 6G) although the final IgG productivity was 5.6g/L, 1.2-fold lower than clone A. Expression analysis of HC and LC mRNA by real-time RTPCR over the course of a 14 day fed-batch culture (Fig 6H and 6I) revealed a steady rise in HC and LC mRNA levels for clone A and clone D between day 0 to day 14. The level of induction for both HC and LC were significantly higher in clone A compared to clone D, correlating with the 2-fold difference in final productivity between these two clones. Interestingly, there was a 3- to 4-fold higher expression in basal mRNA levels for HC and LC in clone A compared to clone D (Fig 6E and 6F) and almost 2-fold difference in basal ERSI (0.145 for clone A compared to 0.078 for clone D, as shown in Fig 6E). On the other hand in clone C, there was a sharp rise in the HC and LC mRNA levels between day 6 and 8 which paralleled with sharp rise in the QP with low IVCC (Fig 6D) and simultaneous induction of p-PERK (Fig 6A), resulting in a temporary halt in the translation by induction of p-eIF2α, which correlated well with a drop in the mRNA levels by day 10 for both chains (Fig 6H and 6I). Despite the high levels of HC and LC mRNA expression in clone C, the final productivity was lower (5.6g/L) than that of clone A (6.9g.L). A similar set of events could be seen in clone B, where sustained activation of PERK is observed. This suggests that PERK activation acts as a regulator to transiently halt protein synthesis. PERK activation coincides with a rapid rise in QP at low IVCC between day 8 and 10. Increased p-PERK activity results in an inhibition of protein translation that reduces the rate of unfolded nascent peptides entering the ER and so decreases the burden on the system. Interestingly, cells with an elevated basal ER stress tend towards higher titers [36]. As an elevated ERSI also correlated with higher sXBP1, BIP, P58^IPK^ and PDI expression, it is likely that these cells are geared toward enhanced folding capacity and therefore better able to cope with unfolded proteins without necessitating slowed translation or induction of apoptosis, which are hallmarks of activation of the PERK pathway (e.g. clone A). In other words, expression of different chaperones such as BiP, PDI and P58^IPK^, driven by the potent transcription factor sXBP1, and the simultaneous down regulation of p-PERK by P58^IPK^ may act as key signaling events that drived efficient secretion of IgG.

## Conclusions

The RXG reporter described herein detects XBP1 splicing upon activation of ER stress, which can be triggered by accumulation of unfolded nascent peptides. Being a potent transactivator, sXBP1 drives the expression of calnexin and calreticulin, essential for proper glycosylation [37], as well as a number of ER chaperones that aid in protein folding. Our experimental results suggest that the up regulation of the transactivator sXBP1 and simultaneous downregulation of p-PERK are key elements for driving high level expression of mAb in stable manufacturing cell lines (Figs 4B and 6A). However, we also established a correlation between high QP with low IVCC by day 8—day 10 in the course of fed-batch culture that results in sustained expression of phosphorylated PERK and simultaneous overexpression of p-eIF2α, which possibly impose intermittent translation attenuation causing lower final IgG productivity despite the high levels of HC and LC mRNA. In other words, PERK activation acts as a regulator to halt protein synthesis transiently to allow proper folding of the nascent peptides accumulated in the ER. We convincingly established that P58^IPK^ inducible by sXBP1[18] actively exerts its dual function by driving the inhibition of p-PERK [34] at an early stage in production kinetics and simultaneously enables proper folding of the nascent IgG molecules resulting in high levels of IgG secretion.

**Fig 7:**
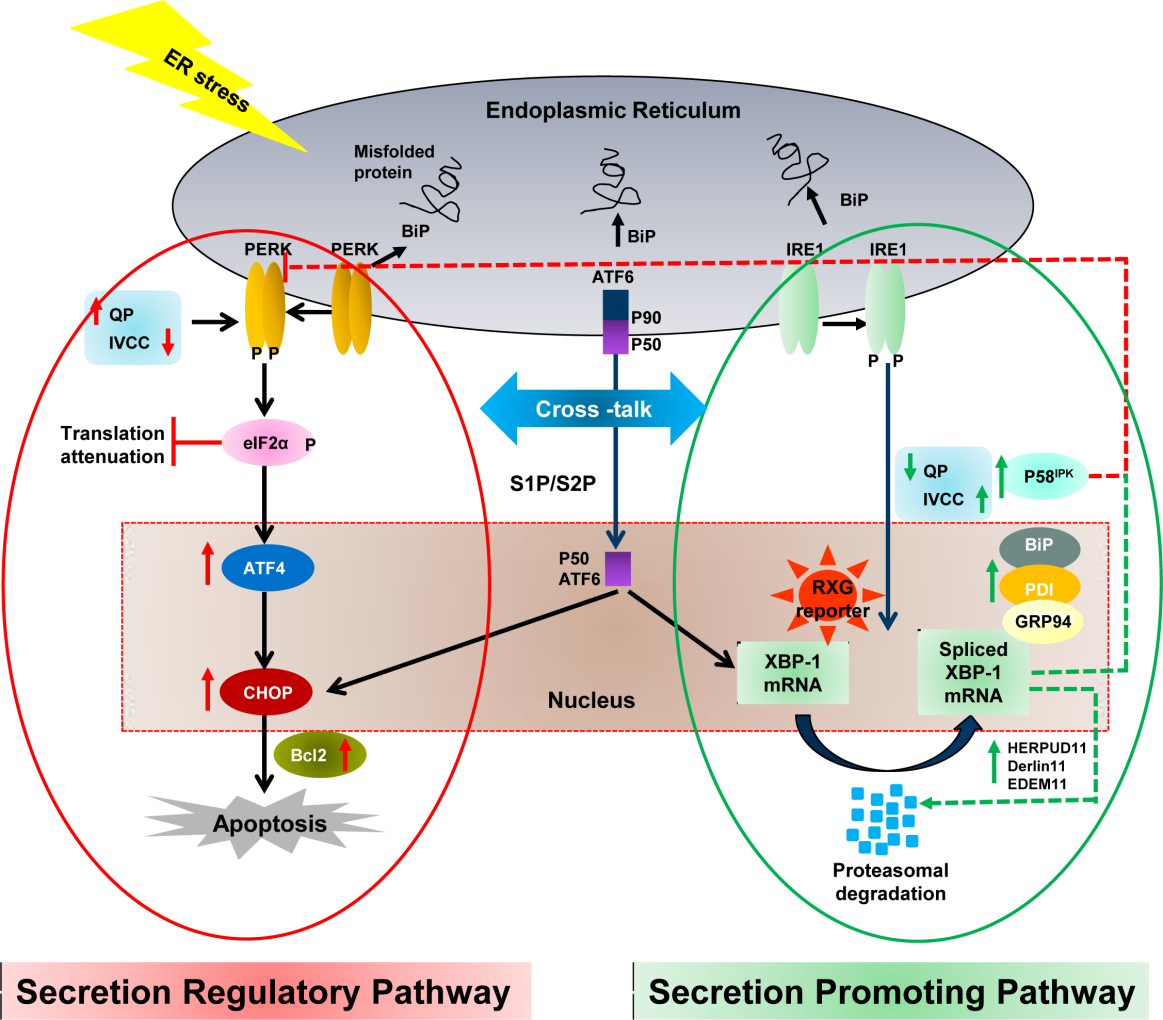
Key elements of ER stress pathway responsible for efficient IgG secretion. Schematic representation of secretion inhibitory and secretion promoting roles regulated by PERK and IRE1 pathway proteins. By inhibiting the PERK pathway, cells with high P58^IPK^ levels signal the activation of the IRE1 pathway and overexpression of protein chaperones (BiP, GRP94 and PDI) necessary to cope with expression levels of high producing cells.

By utilizing the dual fluorescent reporter paired with a thorough biochemical analyses of key ER stress proteins, we were able to distinguish the signaling events associated with terminal and pro-survival characteristics of UPR activation (high levels of IgG expression). In both cases, ER stress was efficiently detected by the reporter. However, in the case of cell treatments with Tm or Tg, there was a moderate induction of p-PERK in addition to XBP1 splicing (Fig 2A and 2B) while the levels of inducible chaperone P58^IPK^ remained unaltered. In contrast, in cells with a high level of IgG secretion, P58^IPK^ was highly induced and concomitant with downregulation of p-PERK. Our data suggests that P58^IPK^ may function to inhibit p-PERK and its downstream signaling events leading to the modulation of UPR as has been previously reported [4, 38]. If P58^IPK^ functions in this way, this molecule may provide a means for cross talk between the PERK and the IRE1 pathways to maintain a coordination of ER stress pathways in the ER space and aid efficient IgG secretion (Fig 7).

In the course of using the dual fluorescent reporter combined with the analysis of the ER stress pathway, we clearly demonstrated the existence of distinct cellular machineries represented by the PERK and IRE1 pathway proteins playing contrasting roles in the inhibition and promotion of secretion; with the potential to impact IgG expression. Interestingly, P58^IPK^ overexpression was observed in high producing cells and may play a critical role in cross-talk between these two pathways enabling a feedback loop by effective inhibition of p-PERK. This feedback inhibition imposed by P58^IPK^ is closely tied to QP and IVCC of IgG expressing cells (Fig 7). A state of homeostasis between synthesis of nascent peptides, activation of different chaperones in the ER induced by the transactivator sXBP1, and cross-talk between key UPR pathway proteins influence efficient IgG secretion. Additionally, we illustrated the utility of this reporter in the clone selection process, which can be used as a tool to enrich the frequency of high titer clones in a typical cell line development campaign through the early detection of ER stress mediated by sXBP1. In light of this study it will be interesting to evaluate how the ER stress is handled by cells while expressing bispecific or highly engineered molecules compared to conventional mAbs and accordingly devise engineering strategies for improved production of these multi-specific bio-therapeutic proteins.

## Supporting information

S1 Fig Confirmation of activation of unfolded protein response by treatment with Tm and Tg in respect to untreated and vehicle (DMSO) treated controls. (TIF)

## References

[1] Wurm FM. Production of recombinant protein therapeutics in cultivated mammalian cells. Nat Biotechnol. 2004;22(11):1393–8. doi: 10.1038/nbt1026 . 15529164 10.1038/nbt1026

[2] Ellgaard L, Helenius A. Quality control in the endoplasmic reticulum. Nat Rev Mol Cell Biol. 2003;4(3):181–91. doi: 10.1038/nrm1052 . 12612637 10.1038/nrm1052

[3] Schroder M, Kaufman RJ. The mammalian unfolded protein response. Annu Rev Biochem. 2005;74:739–89. doi: 10.1146/annurev.biochem.73.011303.074134 . 15952902 10.1146/annurev.biochem.73.011303.074134

[4] Rutkowski DT, Kaufman RJ. A trip to the ER: coping with stress. Trends Cell Biol. 2004;14(1):20–8. . 14729177 10.1016/j.tcb.2003.11.001

[5] Forman MS, Lee VM, Trojanowski JQ. 'Unfolding' pathways in neurodegenerative disease. Trends Neurosci. 2003;26(8):407–10. doi: 10.1016/S0166-2236(03)00197-8 . 12900170 10.1016/S0166-2236(03)00197-8

[6] McCullough KD, Martindale JL, Klotz LO, Aw TY, Holbrook NJ. Gadd153 sensitizes cells to endoplasmic reticulum stress by down-regulating Bcl2 and perturbing the cellular redox state. Molecular and cellular biology. 2001;21(4):1249–59. doi: 10.1128/MCB.21.4.1249-1259.2001 ; PubMed Central PMCID: PMCPMC99578. 11158311 10.1128/MCB.21.4.1249-1259.2001 PMC99578

[7] Paschen W, Frandsen A. Endoplasmic reticulum dysfunction—a common denominator for cell injury in acute and degenerative diseases of the brain? J Neurochem. 2001;79(4):719–25. . 11723164 10.1046/j.1471-4159.2001.00623.x

[8] Szegezdi E, Logue SE, Gorman AM, Samali A. Mediators of endoplasmic reticulum stress-induced apoptosis. EMBO Rep. 2006;7(9):880–5. doi: 10.1038/sj.embor.7400779 ; PubMed Central PMCID: PMCPMC1559676. 16953201 10.1038/sj.embor.7400779 PMC1559676

[9] Cudna RE, Dickson AJ. Endoplasmic reticulum signaling as a determinant of recombinant protein expression. Biotechnology and bioengineering. 2003;81(1):56–65. doi: 10.1002/bit.10445 . 12432581 10.1002/bit.10445

[10] Oyadomari S, Mori M. Roles of CHOP/GADD153 in endoplasmic reticulum stress. Cell Death Differ. 2004;11(4):381–9. Epub 2003/12/20. doi: 10.1038/sj.cdd.4401373 . 14685163 10.1038/sj.cdd.4401373

[11] Walter P, Ron D. The unfolded protein response: from stress pathway to homeostatic regulation. Science. 2011;334(6059):1081–6. Epub 2011/11/26. doi: 10.1126/science.1209038 . 22116877 10.1126/science.1209038

[12] Ye J, Rawson RB, Komuro R, Chen X, Dave UP, Prywes R, et al ER stress induces cleavage of membrane-bound ATF6 by the same proteases that process SREBPs. Mol Cell. 2000;6(6):1355–64. . 11163209 10.1016/s1097-2765(00)00133-7

[13] Yoshida H, Okada T, Haze K, Yanagi H, Yura T, Negishi M, et al ATF6 activated by proteolysis binds in the presence of NF-Y (CBF) directly to the cis-acting element responsible for the mammalian unfolded protein response. Molecular and cellular biology. 2000;20(18):6755–67. ; PubMed Central PMCID: PMCPMC86199. 10958673 10.1128/mcb.20.18.6755-6767.2000 PMC86199

[14] Yoshida H, Matsui T, Yamamoto A, Okada T, Mori K. XBP1 mRNA is induced by ATF6 and spliced by IRE1 in response to ER stress to produce a highly active transcription factor. Cell. 2001;107(7):881–91. Epub 2002/01/10. . 11779464 10.1016/s0092-8674(01)00611-0

[15] Harding HP, Calfon M, Urano F, Novoa I, Ron D. Transcriptional and translational control in the Mammalian unfolded protein response. Annual review of cell and developmental biology. 2002;18:575–99. Epub 2002/07/27. doi: 10.1146/annurev.cellbio.18.011402.160624 . 12142265 10.1146/annurev.cellbio.18.011402.160624

[16] Calfon M, Zeng H, Urano F, Till JH, Hubbard SR, Harding HP, et al IRE1 couples endoplasmic reticulum load to secretory capacity by processing the XBP-1 mRNA. Nature. 2002;415(6867):92–6. Epub 2002/01/10. doi: 10.1038/415092a . 11780124 10.1038/415092a

[17] Wang XZ, Harding HP, Zhang Y, Jolicoeur EM, Kuroda M, Ron D. Cloning of mammalian Ire1 reveals diversity in the ER stress responses. The EMBO journal. 1998;17(19):5708–17. Epub 1998/10/02. doi: 10.1093/emboj/17.19.5708 ; PubMed Central PMCID: PMCPMC1170899. 9755171 10.1093/emboj/17.19.5708 PMC1170899

[18] Lee AH, Iwakoshi NN, Glimcher LH. XBP-1 regulates a subset of endoplasmic reticulum resident chaperone genes in the unfolded protein response. Molecular and cellular biology. 2003;23(21):7448–59. doi: 10.1128/MCB.23.21.7448-7459.2003 ; PubMed Central PMCID: PMCPMC207643. 14559994 10.1128/MCB.23.21.7448-7459.2003 PMC207643

[19] Gass JN, Gifford NM, Brewer JW. Activation of an unfolded protein response during differentiation of antibody-secreting B cells. J Biol Chem. 2002;277(50):49047–54. doi: 10.1074/jbc.M205011200 . 12374812 10.1074/jbc.M205011200

[20] Gunn KE, Brewer JW. The unfolded protein response in marginal zone and follicular zone B cells. Faseb J. 2003;17(7):C209–C.

[21] Reimold AM, Iwakoshi NN, Manis J, Vallabhajosyula P, Szomolanyi-Tsuda E, Gravallese EM, et al Plasma cell differentiation requires the transcription factor XBP-1. Nature. 2001;412(6844):300–7. doi: 10.1038/35085509 . 11460154 10.1038/35085509

[22] Iwakoshi NN, Lee AH, Vallabhajosyula P, Otipoby KL, Rajewsky K, Glimcher LH. Plasma cell differentiation and the unfolded protein response intersect at the transcription factor XBP-1. Nat Immunol. 2003;4(4):321–9. doi: 10.1038/ni907 12612580 10.1038/ni907

[23] van Anken E, Romijn EP, Maggioni C, Mezghrani A, Sitia R, Braakman I, et al Sequential waves of functionally related proteins are expressed when B cells prepare for antibody secretion. Immunity. 2003;18(2):243–53. doi: 10.1016/S1074-7613(03)00024-4 12594951 10.1016/s1074-7613(03)00024-4

[24] Tirosh B, Iwakoshi NN, Glimcher LH, Ploegh HL. XBP-1 specifically promotes IgM synthesis and secretion, but is dispensable for degradation of glycoproteins in primary B cells. J Exp Med. 2005;202(4):505–16. doi: 10.1084/jem.20050575 ; PubMed Central PMCID: PMCPMC2212843. 16103408 10.1084/jem.20050575 PMC2212843

[25] Zhang K, Wong HN, Song B, Miller CN, Scheuner D, Kaufman RJ. The unfolded protein response sensor IRE1α is required at 2 distinct steps in B cell lymphopoiesis. The Journal of Clinical Investigation. 2005;115(2):268–81. doi: 10.1172/JCI21848 15690081 10.1172/JCI21848 PMC546421

[26] Tigges M, Fussenegger M. Xbp1-based engineering of secretory capacity enhances the productivity of Chinese hamster ovary cells. Metab Eng. 2006;8(3):264–72. doi: 10.1016/j.ymben.2006.01.006 . 16635796 10.1016/j.ymben.2006.01.006

[27] Ku SC, Toh PC, Lee YY, Chusainow J, Yap MG, Chao SH. Regulation of XBP-1 signaling during transient and stable recombinant protein production in CHO cells. Biotechnology progress. 2010;26(2):517–26. Epub 2009/11/26. doi: 10.1002/btpr.322 . 19938059 10.1002/btpr.322

[28] Cain K, Peters S, Hailu H, Sweeney B, Stephens P, Heads J, et al A CHO cell line engineered to express XBP1 and ERO1-Lalpha has increased levels of transient protein expression. Biotechnology progress. 2013;29(3):697–706. doi: 10.1002/btpr.1693 . 23335490 10.1002/btpr.1693

[29] Becker E, Florin L, Pfizenmaier K, Kaufmann H. An XBP-1 dependent bottle-neck in production of IgG subtype antibodies in chemically defined serum-free Chinese hamster ovary (CHO) fed-batch processes. J Biotechnol. 2008;135(2):217–23. doi: 10.1016/j.jbiotec.2008.03.008 . 18448183 10.1016/j.jbiotec.2008.03.008

[30] Du Z, Treiber D, McCoy RE, Miller AK, Han M, He F, et al Non-invasive UPR monitoring system and its applications in CHO production cultures. Biotechnology and bioengineering. 2013;110(8):2184–94. doi: 10.1002/bit.24877 . 23436541 10.1002/bit.24877

[31] Kober L, Zehe C, Bode J. Development of a novel ER stress based selection system for the isolation of highly productive clones. Biotechnology and bioengineering. 2012;109(10):2599–611. Epub 2012/04/19. doi: 10.1002/bit.24527 . 22510960 10.1002/bit.24527

[32] Lytton J, Westlin M, Hanley MR. Thapsigargin inhibits the sarcoplasmic or endoplasmic reticulum Ca-ATPase family of calcium pumps. J Biol Chem. 1991;266(26):17067–71. Epub 1991/09/15. . 1832668

[33] DuRose JB, Tam AB, Niwa M. Intrinsic capacities of molecular sensors of the unfolded protein response to sense alternate forms of endoplasmic reticulum stress. Mol Biol Cell. 2006;17(7):3095–107. doi: 10.1091/mbc.E06-01-0055 ; PubMed Central PMCID: PMCPMC1483043. 16672378 10.1091/mbc.E06-01-0055 PMC1483043

[34] van Huizen R, Martindale JL, Gorospe M, Holbrook NJ. P58IPK, a novel endoplasmic reticulum stress-inducible protein and potential negative regulator of eIF2alpha signaling. J Biol Chem. 2003;278(18):15558–64. doi: 10.1074/jbc.M212074200 . 12601012 10.1074/jbc.M212074200

[35] Evans K, Albanetti T, Venkat R, Schoner R, Savery J, Miro-Quesada G, et al Assurance of monoclonality in one round of cloning through cell sorting for single cell deposition coupled with high resolution cell imaging. Biotechnology progress. 2015;31(5):1172–8. doi: 10.1002/btpr.2145 26195345 10.1002/btpr.2145 PMC5054913

[36] Prashad K, Mehra S. Dynamics of unfolded protein response in recombinant CHO cells. Cytotechnology. 2015;67(2):237–54. doi: 10.1007/s10616-013-9678-8 ; PubMed Central PMCID: PMCPMC4329310. 24504562 10.1007/s10616-013-9678-8 PMC4329310

[37] Hammond C, Braakman I, Helenius A. Role of N-linked oligosaccharide recognition, glucose trimming, and calnexin in glycoprotein folding and quality control. Proc Natl Acad Sci U S A. 1994;91(3):913–7. ; PubMed Central PMCID: PMCPMC521423. 8302866 10.1073/pnas.91.3.913 PMC521423

[38] Chakrabarti A, Chen AW, Varner JD. A review of the mammalian unfolded protein response. Biotechnology and bioengineering. 2011;108(12):2777–93. Epub 2011/08/03. doi: 10.1002/bit.23282 ; PubMed Central PMCID: PMCPMC3193940. 21809331 10.1002/bit.23282 PMC3193940

