# Supplementary figures and images for: Development of a fluorescent reporter system for monitoring ER stress in Chinese hamster ovary cells and its application for therapeutic protein production
Source: PLoS One. 2017 Aug 23;12(8):e0183694. doi: 10.1371/journal.pone.0183694 (PMC5568292; doi:10.1371/journal.pone.0183694)

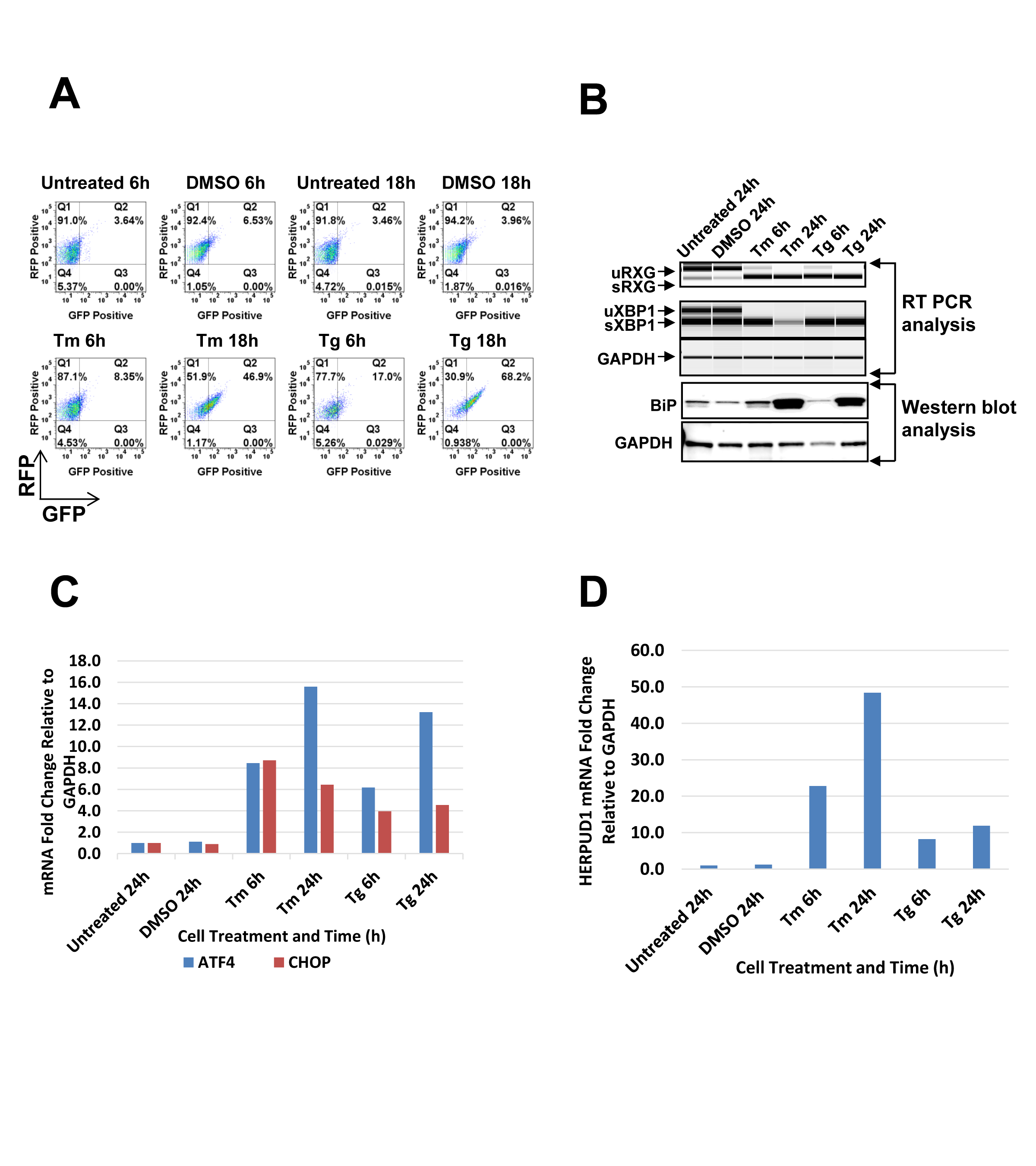

Supplement: S1 Fig — (TIF) [file pone.0183694.s001.tif]
